# The Neuroprotective Effects of Cannabis-Derived Phytocannabinoids and Resveratrol in Parkinson’s Disease: A Systematic Literature Review of Pre-Clinical Studies

**DOI:** 10.3390/brainsci11121573

**Published:** 2021-11-28

**Authors:** Samay Prakash, Wayne G. Carter

**Affiliations:** Royal Derby Hospital Centre, School of Medicine, University of Nottingham, Derby DE22 3DT, UK; mzysp8@nottingham.ac.uk

**Keywords:** cannabinoids, cannabis-derived phytocannabinoids, neuroprotection, resveratrol, Parkinson’s disease

## Abstract

Currently, there are no pharmacological treatments able to reverse nigral degeneration in Parkinson’s disease (PD), hence the unmet need for the provision of neuroprotective agents. Cannabis-derived phytocannabinoids (CDCs) and resveratrol (RSV) may be useful neuroprotective agents for PD due to their anti-oxidative and anti-inflammatory properties. To evaluate this, we undertook a systematic review of the scientific literature to assess the neuroprotective effects of CDCs and RSV treatments in pre-clinical in vivo animal models of PD. The literature databases MEDLINE, EMBASE, PsychINFO, PubMed, and Web of Science core collection were systematically searched to cover relevant studies. A total of 1034 publications were analyzed, of which 18 met the eligibility criteria for this review. Collectively, the majority of PD rodent studies demonstrated that treatment with CDCs or RSV produced a significant improvement in motor function and mitigated the loss of dopaminergic neurons. Biochemical analysis of rodent brain tissue suggested that neuroprotection was mediated by anti-oxidative, anti-inflammatory, and anti-apoptotic mechanisms. This review highlights the neuroprotective potential of CDCs and RSV for in vivo models of PD and therefore suggests their potential translation to human clinical trials to either ameliorate PD progression and/or be implemented as a prophylactic means to reduce the risk of development of PD.

## 1. Introduction

Parkinson’s disease (PD) is a neurodegenerative motor disorder that primarily affects the elderly. PD is progressive, and patients typically display a clinical triad of motor symptoms that are postural rigidity, bradykinesia, and resting tremor [1]. However, patients with PD may also display non-motor symptomology and overlap of signs and symptoms with atypical Parkinsonian syndromes such as multiple system atrophy (MSA) and dementia with Lewy bodies, which renders absolute diagnosis challenging [2]. Approximately 1% of the population over the age of 70 and an estimated 6.2 million individuals worldwide are affected by PD, and this is expected to increase yearly in line with a burgeoning geriatric population [3,4,5]. PD is characterized histopathologically by the loss of dopaminergic neurons within the substantia nigra pars compacta (SNpc) and accumulation of protein aggregates including α-synuclein within Lewy-bodies (LBs) [6,7,8,9]. The oligomeric and aggregated forms of α-synuclein can be neurotoxic and can promote loss of dopaminergic neurons [6,7,8,9]. PD is primarily an idiopathic disease, for which age is the major risk factor [10]. However, other risk factors associated with lifestyle and environmental exposures, such as alcohol intake and pesticide exposures have also been proposed; although a causal relationship between these and disease pathogenesis has yet to be clearly established [11,12]. Genetic vulnerability to PD has been observed in a minority of PD cases (10–15%) via rare familial mutants, including those in α-synuclein that trigger early onset Parkinsonian phenotypes, and other inheritable gene mutations that may contribute to cellular damage, oxidative stress, and neuroinflammation via microglial activation [13,14].

At present, there are no curative treatments for PD, just pharmacotherapy to manage symptoms. The first-line treatment, levodopa, is employed to supplement dopamine levels to mitigate the loss of dopaminergic innervations [15,16,17]. L-dopa administration is usually accompanied by a decarboxylase inhibitor such as carbidopa, to limit its peripheral metabolism [17,18,19]. Chronic administration of L-dopa is associated with dyskinesias and reduced drug efficacy, thus making symptom management increasingly difficult with disease progression [17,18]. Similarly, there are no standard neuroprotective medications in PD treatment able to cease disease progression, although monoamine oxidase-B inhibitors (MAO-B) or dopamine receptor agonists may have neuroprotective as well as symptomatic effects [17,18,19]. 

### 1.1. Neurotoxin and Genetic Models of PD

Although the majority of cases of PD are idiopathic, environmental toxins can rapidly induce Parkinsonian phenotypes. Hence, models of PD have invariably utilized chemical neurotoxins that trigger damage to the SNpc, reduce dopamine levels, induce mitochondrial damage, redox stress, and neuroinflammation, and thereby reproduce many of the elements of PD pathology. Commonly used chemical neurotoxins include rotenone, 1-methyl-4-phenyl-1,2,3,6-tetrahydropyridine (MPTP), 6-hydroxydopamine (6-OHDA), and paraquat [20,21,22]. 

Rotenone is an agricultural pesticide that can cross the blood-brain barrier (BBB) and enter dopaminergic neurons independently of DA transporters and inhibits mitochondrial complex I and associated ATP production via the coupling of the electron transport chain to oxidative phosphorylation. Rotenone also generates redox stress and can induce α-synuclein positive aggregations [20,21,22]. MPTP can cross the BBB where it is metabolized by monoamine oxidase-B (MAO-B) in astrocytes into the potent neurotoxin, 1-methyl-4-phenylpyridimium (MPP+), which behaves as a structural analogue to dopamine and enters dopaminergic neurons via DA transporters. MPP+ is also a mitochondrial inhibitor and can trigger cellular redox stress [20,21,22]. 6-OHDA is a dopamine analogue with a high affinity for dopamine transporters but without BBB penetrance. Hence, 6-OHDA is usually administered via unilateral intracerebral injection into the median foramen bundle or SNpc. Its neurotoxic effects are attributed to its accumulation in the cytosol and mitochondria of neurons, leading to redox stress, and also in part via inhibition of mitochondrial complex I activity. Additionally, the administration of 6-OHDA induces neuroinflammation [20,21,22]. Paraquat is a divalent cationic herbicide similar in structure to MPP+ that is also capable of damaging mitochondria and the induction of redox stress [20,21,22]. Reserpine blocks neurotransmitter uptake into vesicles and can establish the akinetic symptomatology of PD [23]. Lipopolysaccharide (LPS), an endotoxin from the outer membrane of Gram-negative bacteria, has been used as a toxin to stimulate neuroinflammation, via the production of pro-inflammatory cytokines and generation of reactive oxygen species (ROS) from microglia [21]. 

Despite the majority of PD being sporadic, genetic animal models of PD have proved useful in establishing a causal relationship between certain genes and the development of familial PD [21,22,24]. Genes implicated in familial PD development include autosomal dominant α-synuclein mutants, such as the A30P, A53T, and E46K mutants. Autosomal dominant mutants of *LRRK2*, as well as autosomal recessive mutations in *Parkin*, *DJ-1* and *PINK* 1, can also reproduce key features of PD pathology [21,22,24].

### 1.2. Cannabis-Derived Cannabinoids and Resveratrol as Neuroprotective Agents

There has been a recent upsurge in the potential use of dietary polyphenols as neuroprotective agents in neurodegenerative diseases including PD [25,26,27,28] [Supplementary Figure S1]. *Cannabis sativa* is a natural herbaceous plant historically consumed or smoked for recreational and therapeutic purposes [29]. Tetrahydrocannabinol (THC) and cannabidiol (CBD) are major phytocannabinoid constituents of the cannabis plant and act on cannabinoid receptors [29,30]. *Cannabis sativa* also contains a plethora of phytochemicals including the minor phytocannabinoids β-caryophyllene (BCP) and tetrahydrocannabivarin (THCV) [31,32]. BCP is a component of cannabis essential oil and essential oils from cinnamon, black pepper, and oregano [31]. These compounds may display neuroprotective activity via cannabinoid receptor-2 (CB2) agonistic effects, and without exhibiting the psychoactive effects of THC [30,31,32,33]. BCP exerts anti-inflammatory activity via activation of members of the nuclear receptor family of peroxisome proliferated activator receptors (PPARs) [33,34].

Resveratrol (RSV) is a natural stilbene present in several dietary foodstuffs including berries and grapes, as well as red wine, for which consumption of RSV is associated with a number of purported health benefits [27,35,36]. RSV is also a component of the herbaceous root *Polygonum Cuspidatum*, used in traditional Chinese herbal medicine, with recorded anti-oxidative and anti-inflammatory effects [37]. 

CBD, THC, THCV, and RSV are polyphenols and therefore may provide useful neuroprotective activity due to their antioxidant free radical scavenging ability. The structures of these compounds and BCP are shown in Table 1. The neuro-modulatory potential of these cannabis-derived phytocannabinoids (CDCs) and RSV overlap, such that both can be consumed through recreational use as cannabis and alcoholic drinks including red wine, respectively [26,27]. Therefore, a systematic literature review was undertaken to appraise the experimental literature that has considered the potential neuroprotective effects of these CDCs and RSV in pre-clinical animal studies of PD. 

## 2. Materials and Methods

A systematic review of the literature was conducted following the guidance of the Preferred Reporting Items for Systematic Reviews and Meta-Analyses (PRISMA) [38].

### 2.1. Search Strategy 

An electronic literature search was performed using MEDLINE, EMBASE, PsychINFO, PubMed, and Web of Science Core Collection to retrieve pre-clinical experimental studies investigating the neuroprotective effects of these CDCs and RSV in animal models of PD. A combination of Boolean operators (AND/OR) and field tags (multipurpose) were employed for the following major search terms: Parkinson’s disease; Parkinson*; cannab*; tetrahydrocannabinol; cannabidiol; β-caryophyllene; tetrahydrocannabivarin; resveratrol; in vivo; pre-clinical; animal, primate, monkey, rodent, mice, mouse, rat. The full OVID and PubMed search strategy is provided as Supplementary Data S2. The addition of hand-searched studies from references and bibliographies of related publications was performed secondarily.

### 2.2. Eligibility Criteria 

All search results (*n* = 1034) were exported into EndNote (reference manager) for removal of duplicate publications and text analysis with respect to predefined eligibility criteria. Included articles were original, full-text publications, published in English between database inception and January 2020 that investigated the neuroprotective effects of CDCs or RSV in PD animal models in vivo, with no restriction on age, dosing, length of study or outcome measures. Studies were excluded if the experiment was performed on non-rodent animals or artificial CDCs or RSV derivatives were used. 

### 2.3. Data Acquisition and Analysis 

A total of 18 eligible publications were retrieved using the University of Nottingham Library (NUsearch) facility or via an inter-library loan request from the British Library. The following variables were extracted to an Excel data spreadsheet: author(s), year of publication, the aim of the study, population, intervention, dosing, length of study, outcome measures and results in order to generate a literature review. Both study authors reviewed the included papers, independently considered the data extraction, and discussed all papers that were included in the final review. Due to the qualitative nature and heterogeneity of some of the study outcomes, a meta-analysis was not performed.

For methodological quality assessment, the Systematic Review Centre for Laboratory animal Experimentation (SYRCLE) risk of bias tool was considered (Supplementary Table S1), which has been adapted in accordance with methodology used in animal studies [39]. 

## 3. Results

**Figure 1:**
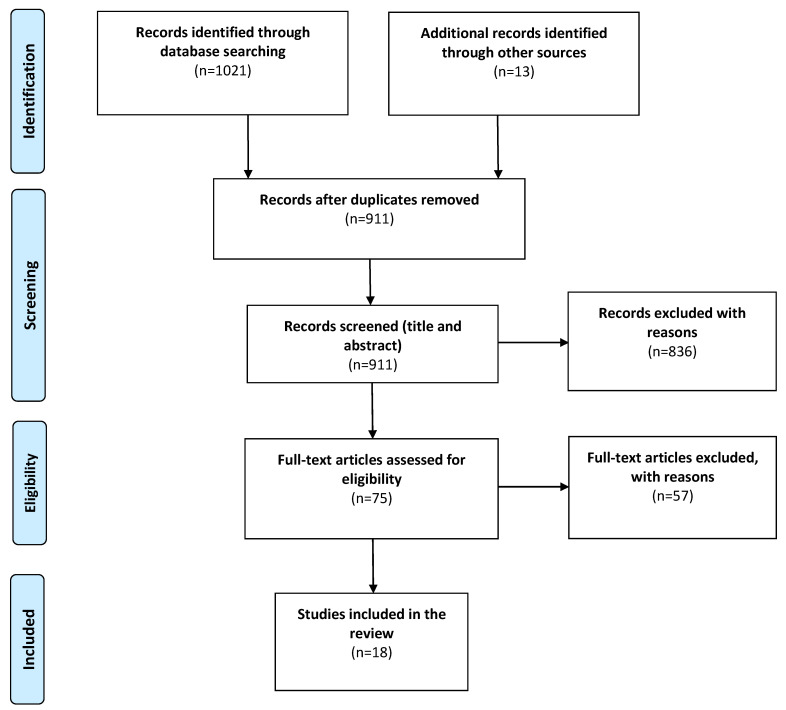
Preferred reporting items for systematic reviews and meta-analyses (PRISMA) flow chart detailing the stages of study retrieval and selection [38].

The preliminary data search generated 1021 results, reduced to 898 following the removal of duplicates. The addition of 13 hand-searched studies from references and bibliographies of related publications, resulted in a total of 911 papers, which were screened with respect to their titles and abstracts. Of these 911 papers, 836 did not meet the pre-defined inclusion criteria and were excluded for the following reasons: irrelevant, performed on non-rodent models, in vitro studies, focused on other neurodegenerative diseases, full-text inaccessibility and lack of specificity. Full-text articles were read in full for 75 studies, of which 57 were removed on the grounds of investigating synthetic cannabinoids, cannabinoid receptor agonists or resveratrol-related compounds, and therefore did not meet the eligibility criteria. The remaining 18 studies fulfilled the eligibility criteria and were included in the final analysis of this review (Figure 1). The majority of studies investigated RSV (*n* = 12) and the remaining studies CDCs (*n* = 6); specifically, tetrahydrocannabinol (THC) (*n* = 2), cannabidiol (CBD) (*n* = 2), tetrahydrocannabivarin (THCV) (*n* = 1), and β-caryophyllene (BCP) (*n* = 2). 

### 3.1. Study Characteristics 

Six strains from two different rodent species were assessed: Wistar rats (*n* = 252), Sprague Dawley rats (*n* = 183), C57BL/6 mice (*n* = 156), Balb/C mice (*n* = 60), Swiss Albino mice (*n* = 42), and A53T-α synuclein mice (*n* = 40). Two studies did not report the strain of mice investigated (*n* = 39). An average of 43 rodents were used per study, with a median of 41 and a range of 24–60. All rodents were male. The weight of rats and mice ranged from 180–350 g and 20–35 g, respectively.

### 3.2. Dosing

A number of different neurotoxic models were used to produce a Parkinsonian phenotype, and these have been divided by their intervention group (BCP, CBD, THC, THCV, and RSV), and then neurotoxin model and ascending year of study (Table 2) [40,41,42,43,44,45,46,47,48,49,50,51,52,53,54,55,56,57]. 6-Hydroxydopamine (6-OHDA) was administered to rats (*n* = 6), of which four studies used a single administration with a dose of 8–15 μg, whilst two studies gave daily injections over 14 days of 8 μg and 200 μg [40,41,42,43,44,45]. Unilateral 6-OHDA lesions were induced via intracerebroventricular injection, striatal injection, medial forebrain bundle injection or intraperitoneal injection [40,41,42,43,44,45]. 1-Methyl-4-phenyl-1,2,3,6-tetrahydropyridine (MPTP) was administered to mice at 20 or 30 mg/kg intraperitoneally (*n* = 6), acutely (4 doses over 8 h) or daily for 4–7 days [52,53,54,55,56,57]. Rats were dosed with rotenone (*n* = 3) either daily or every other day, at 1.5–2.5 mg/kg by intraperitoneal or subcutaneous routes, for a duration of 21–35 days [47,48,50]. Reserpine was administered to rats (*n* = 1) at 1 mg/kg subcutaneously twice over a 5 day period [49]. Haloperidol was administered intraperitoneally to mice at 1 mg/kg (*n* = 1) once daily for 18 days [46]. A single study used 5 μg of lipopolysaccharide (LPS) via intracerebroventricular injection over 14 days in a subset of mice [41], and one study did not utilize a neurotoxin, but instead assessed the effects of RSV on transgenic A53T α-synuclein mice that naturally developed α-synuclein aggregates at 9–16 months of age [51].

Six of the 18 studies investigated the neuroprotective effects of four different CDCs (BCP, CBD, THC, and THCV) [40,41,46,47,49,57]. Rodents in two studies were given THC (intraperitoneally or subcutaneously) at a dose of either 3, 10, or 20 mg/kg daily for 14–18 days [40,46]. THCV was administered intraperitoneally in one study (2 mg/kg, daily for either 14 days or 14 days post-lesion) [41]. CBD was administered to rats in two studies at 0.5–5 mg/kg, intraperitoneally, for 3–14 days [40,49]. BCP was administered at 10 mg/kg intraperitoneally or by oral gavage for 5 days in mice, or at a higher dose of 50 mg/kg for 28 days in rats [47,57]. Twelve studies investigated the neuroprotective effect of RSV using a dosing range of 10–100 mg/kg per day for 1 to 10 weeks [42,43,44,45,48,50,51,52,53,54,55,56]. The most common route of administration was orally (*per os* and oral gavage) but intragastric gavage, intraperitoneal and intravenous routes were also used [42,43,44,45,48,50,51,52,53,54,55,56]. 

The study outcomes have been divided into behavioral, cellular, and molecular changes (Table 2).

### 3.3. Behavioral Outcomes

Fourteen studies utilized an array of behavioral outcome measures to observe cognitive and motor changes in rodent PD models and assessed whether these impairments were mitigated by the administration of CDCs or RSV. The results of these studies are listed in Table 3 and have been divided by their intervention group (BCP, CBD, THC, THCV, and RSV), and then ascending year of study. 

#### 3.3.1. Open Field Testing and Movement 

A single study evaluated the neuroprotective effect of CBD in a risperidone-induced PD rat model using an open field circular arena and concluded that the locomotor activity of the risperidone group was significantly lower than the control group [49]. CBD treatment ameliorated risperidone-induced memory deficits but not locomotor activity, although oral movements as vacuous chewing were also reduced after treatment with CBD [49].

Motor activity was also examined using a computer-aided actimeter (CAA) and reported a significant increase in distance travelled and mean velocity in THCV-treated rodents when compared to the PD model [41].

PD model rodents displayed reduced velocity, rearing, and distance travelled and these impairments were significantly improved by RSV treatment [45,50,52,54]. By contrast, a single study reported increased velocity in a PD model (A53T α-synuclein mice), but that was returned to control levels after treatment with RSV [51].

Rotational (circling) behavior was examined in three studies and demonstrated the benefit of RSV treatment to reduce apomorphine-induced circling behavior [42,43,44]. 

#### 3.3.2. Rotarod and Grasp Strength 

A rotarod test was used to assess grip strength and balance. Measurements were based on the time rodents remained on a rotating metal rod before falling off. Across six studies that used this method, all investigated the effects of RSV, but with slightly different experimental methods. The size of the rods ranged from 0.75–6 cm in diameter and maximum rotational speed varied from 12–20 rotations per minute. All studies showed reduced retention time on the rotarod in PD model groups relative to control groups [43,45,48,50,54,56]. One study also assessed grasp strength, measured by having rodents hold onto a horizontal bar over six trials. In comparison with the control group, the PD model group displayed increased grasp strength, indicating muscle rigidity, and this grasp strength was reduced by RSV administration [56].

#### 3.3.3. Pole and Beam Test

A pole test was used to assess bradykinesia and was performed by placing the rodent at the top of a pole with its head facing upwards. The time that was taken for a rodent to turn around and descend the pole was recorded. Of the three studies that used this measure, the height of the pole varied from 50–55 cm. An average of at least three trials was recorded per study. The beam test is similar, with rodents placed at one end of a narrow beam, and the time taken to reach the other end measured. Rodents within the PD model groups spent a significantly increased time in the beam and pole tests compared to controls, whereas rodents that received BCP both orally and *i.p.*, or RSV, displayed significantly decreased beam and pole test times [51,52,54,57].

#### 3.3.4. Gait Assessment and Stepping Test

Gait assessment was used to monitor a change in stride length by measuring the distances between forepaw prints in rodents. A shortened stride length is observed for rodent models of PD [58]. Provision of oral or *i.p.* BCP or administered RSV countered this reduced stride length [52,57]. A stepping test was also performed in a single study, and the number of adjusting steps taken when forced to walk on one forepaw was recorded. There was a reduction of adjustment steps in the PD model group, which was significantly rectified by RSV administration [43]. 

#### 3.3.5. Catalepsy

Catalepsy, a decrease in movement and inability to correct abnormal posture, was assessed by the bar test and the grid test across four studies. The bar test involved placing a rodent’s hindlimbs on a bench and their forepaws on an elevated horizontal bar, and quantification of the time that they remain in this position. Catalepsy was attenuated in CBD and THC administered groups, resulting in a significantly decreased time spent in the bar test [46,49]. Two studies employed a grid test in which rodents were hung from a vertical grid approximately 0.5 m high. One study measured the time taken for the rodent to initiate corrective movement [48], while the other measured the time taken to fall from the grid [45]. Both studies showed significantly increased catalepsy in PD model groups, and this was significantly rectified after receiving RSV [45,48].

#### 3.3.6. Apomorphine-Induced Circling Behavior

Apomorphine-induced circling behavior was assessed in three studies. Apomorphine, a non-selective DA receptor agonist influences rotational behavior in rodents [59]. Apomorphine was administered subcutaneously to rats and the net number of contralateral rotations was measured over a time course. All studies demonstrated increased rotational behavior in the PD model group of rats, and that RSV significantly decreased the number of rotations [42,43,44]. 

### 3.4. Biochemical and Immunohistochemical Outcomes

Thirteen studies assessed the neuroprotective effect of CDCs or RSV via an assessment of the levels of dopaminergic neurons and dopamine in the striatum of rodents, and related metabolites such as 3,4-dihydroxyphenylacetic acid (DOPAC) and homovanillic acid (HVA) [40,41,42,44,45,47,48,51,52,53,54,55,57]. The results of these studies have been detailed in Table 4 and have been divided by their intervention group (BCP, CBD, THC, THCV, and RSV), and then ascending year of study. The striatal concentration of tyrosine-hydroxylase (TH) markers were quantified in nine studies [40,41,44,45,51,52,53,55,57]. One study showed a significant five-fold decrease in striatal dopamine levels in a 6-OHDA-induced PD model, that was partially restored by THCV administration, although this did not reach significance [41]. The same study showed a significant decrease in SNpc dopaminergic neurons in mice administered LPS, which was significantly restored via administration of THCV or cannabidiol (CBD)-derived drug (HU-308) in interventional groups [41]. There was a substantial decrease in striatal dopamine and DOPAC in 6-OHDA treated mice, in addition to decreased TH-immunostaining and TH mRNA when compared to control groups, and these decreases were significantly improved by administration of THC in interventional groups [40]. TH immunoreactivity levels also showed a significant decrease in two studies using neurotoxin-PD-induced Wistar rats and C57BL/6J mice, which was significantly restored by BCP administration [47,57]. For markers of dopamine loss (DA and DAN) or injury that had declined in PD model groups, all could be significantly restored in interventional groups treated with RSV [42,47,48,53,54]. 

### 3.5. The Effectiveness of CDCs or RSV to Combat Oxidative Stress

Nine studies investigated the effects of CDCs and RSV on oxidative stress in rodent brain tissue [43,44,46,47,48,50,51,54,56] and included neurotoxin models [43,44,46,47,48,50,54,56], and the α-synuclein genetic model of PD [51]. The results of these studies are shown in Table 5 and have been divided by their intervention group (BCP, THC, and RSV), and then ascending year of study. Protein carbonyl content (PCC), nitric oxide (NO) levels, malondialdehyde (MDA) levels, and thiobarbituric acid reactive substances (TBARS) (by-products of lipid peroxidation reactions), were investigated in studies researching the effects of THC, BCP, and RSV. Levels of PCC, NO, MDA, and TBARS were increased in PD model groups relative to controls, and these were significantly attenuated by treatment with THC, BCP, or RSV [43,46,47,48,50,51,54]. Increased ROS and dihydroxybenzoic acid (DHBA) levels are indicative of elevated free radical levels, and these were increased in the PD model groups for three studies investigating the effects of RSV. These increases were significantly attenuated by RSV administration [44,51,56]. 

Endogenous anti-oxidative agents including reduced glutathione (GSH), and the enzymes, superoxide dismutase (SOD), catalase (CAT), glutathione reductase (GR), glutathione peroxidase (GPx), xanthine oxidase (XO), as well as the citric acid cycle enzymes aconitase, citrate synthase (CS), and succinate dehydrogenase (SDH) were investigated as markers of oxidative stress across seven studies that investigated the effects of BCP, THC, and RSV [43,46,47,48,50,51,54]. These studies showed decreased antioxidant capacity in PD model groups, which could be significantly mitigated by BCP, TCH, or RSV administration [43,46,47,48,50,51,54], except for the study of Anandhan et al. (2010) [54] that reported elevated SOD and CAT activities in their PD model. Total antioxidant capacity (T-AOC) was also increased in response to RSV treatment in the intervention group [44]. One study also monitored mitochondrial complex-I (MC-1) activity, which was decreased in the PD model group but significantly increased by RSV treatment [50]. The cellular antioxidant defense was driven by increased Nrf-2 DNA binding activity in the RSV-treated group relative to the PD model group [48]. 

### 3.6. The Effectiveness of CDCs or RSV to Combat Neuroinflammation

Seven studies investigated the effects of RSV and CDCs on neuroinflammation in rodent brain tissue of the striatum and SNpc [41,42,47,48,51,53,57], and included neurotoxin models [42,47,48,53,57], a genetic model [51], as well as specific induction of neuroinflammation via LPS treatment [41]. The results of these studies are summarized in Table 6 and have been divided by their intervention group (BCP, THCV, and RSV), and then ascending year of study. Five studies showed increased markers of microglia and astrocytes activation via quantification of glial fibrillary acidic protein (GFAP) and ionized calcium-binding adaptor molecule 1 (Iba-1) protein or mRNA levels, and these were significantly reduced via administration of THCV, BCP, or RSV [41,47,51,53,57]. Inflammatory protein markers and their complementary mRNA levels were significantly increased in the PD model groups and this was significantly countered with BCP or RSV treatment [42,47,48,51,53,57]. The suppressor of cytokine signaling protein 1 (SOCS-1) was detected in α-synuclein transgenic mice and was significantly upregulated by RSV treatment [53].

### 3.7. The Anti-Apoptotic Effects of CDCs or RSV

Four studies assessed the anti-apoptotic effects of RSV [44,45,48,52], all of which utilized neurotoxin models [44,45,48,52]. A summary of the RSV studies is included in Table 7 and have been divided by the ascending year of study. One study observed upregulation of apoptotic mediators in the PD model group that was decreased by RSV administration, with reduced neuronal apoptosis [44]. Procaspase and activated caspase-3 as key inducers of neuronal apoptosis were assessed by three studies, and all displayed increased caspase levels in PD model groups relative to controls [45,48,52]. Caspase levels were significantly decreased in groups receiving RSV treatment [45,48,52]. Bcl-2-associated X protein (Bax) and other pro-apoptotic regulators from the B-cell lymphoma 2 (Bcl-2) family were upregulated in PD model groups and were significantly reduced by RSV administration [45]. Increased p62 in nuclear factor kappa beta (NF-κβ) induced autophagy and increased acetylated microtubule-associated protein 1A/1B-light chain 3 (LC3-II) were countered with RSV treatment [52]. Increased C/EBP homologous protein (CHOP) and glucose regulated protein (GRP78), both apoptotic markers of endoplasmic reticulum (ER) related oxidative stress, were significantly reduced by RSV treatment [48]. Although CDCs may exert anti-apoptotic effects, this was not investigated in the studies captured in this literature review.

## 4. Discussion

**Figure 2:**
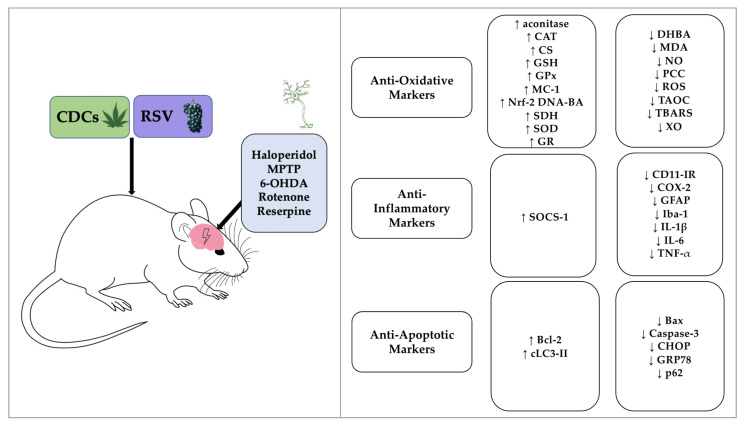
Schematic summary of the neuroprotective effects of CDCs or RSV following induction of PD in rodents. Abbreviations: Bax, Bcl-2-associated X protein; Bcl-2, B-cell lymphoma 2; CAT, catalase; CD11-IR, CD11 immunoreactivity; CDCs, cannabis-derived cannabinoids; CHOP, CCAAT/enhancer binding protein homologous protein; CS, citrate synthase; COX-2, cyclooxygenase-2; DHBA, dihydroxybenzoic acid; GFAP, glial fibrillary acidic protein; GPx, glutathione peroxidase; GR, glutathione reductase; GRP78, glucose-regulated protein 78; GSH, glutathione; 6-OHDA, 6-Hydroxydopamine; Iba-1, ionized calcium-binding adaptor molecule-1; IL-1β, interleukin-1 beta; IL-6, interleukin-6; LC3-II, microtubule-associated protein 1A/1B-light chain 3; MC-1, mitochondrial complex 1; MDA, malondialdehyde;; MPTP, 1-methyl-4-phenyl-1,2,3,6-tetrahydropyridine; NO, nitric oxide; Nrf-2 DNA-BA, Nrf-2 DNA binding activity; PCC, protein carbonyl content; ROS, reactive oxygen species; RSV, resveratrol; SDH, succinate dehydrogenase; SOCS-1, suppressor of cytokine signalling-1; SOD, superoxide dismutase; TAOC, total antioxidant capacity; TBARS, thiobarbituric acid reactive substances; TNF-α, tumor necrosis factor-alpha; XO, xanthine oxidase. ↑, , denotes an increase; ↓, denotes a decrease.

**Figure 3:**
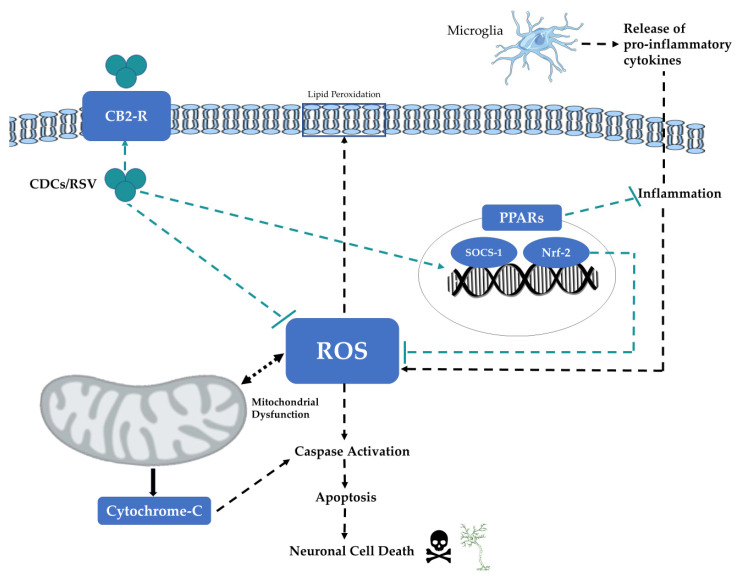
Schematic summary of the potential neuroprotective mechanisms of CDCs or RSV in neurons following induction of PD. Redox stress and the production of reactive oxygen species (ROS) can damage mitochondria and this can further exacerbate ROS production and trigger the release of mitochondrial cytochrome-c and induction of apoptosis. ROS also damages cellular protein targets and induces lipid peroxidation. ROS levels are also elevated in response to inflammatory mediators released by microglia and astrocytes. CDCs and RSV are neuroprotective and scavenge free radicals to reduce the levels of ROS and associated cellular redox stress. CDCs or RSV promotes the activity of PPARs, SOCS-1, and Nrf-2 to reduce inflammation and the induction of ROS.

This literature review considered the neuroprotective effects of certain CDCs and RSV for a range of rodent models of PD. Since degeneration of dopaminergic neurons (in the SNpc) is one of the key pathological hallmarks of PD, quantitative analysis of dopamine levels and dopaminergic neurons was utilized as a primary indicator for neuroprotection. The in vivo rodent studies supported the hypothesis that these agents were neuroprotective against PD and resulted in increased dopamine and dopaminergic neuron levels in response to CDCs or RSV treatment, consistent with a recent meta-analysis [60]. Collectively, the molecular mechanisms associated with neuroprotection reflected anti-oxidative, anti-inflammatory, and anti-apoptotic capabilities (Figure 2 and Figure 3). 

### 4.1. Neuroprotective Effects and Mechanisms

#### 4.1.1. Behavioral Improvements Indicative of Neuroprotection

The reduction in motor and cognitive functions in PD mice models were attributed to neurotoxin-induced dopaminergic neuron loss within the SNpc, resulting in a dysfunctional striatal pathway and overstimulation of GABAergic neurons innervating the thalamus [61]. Reduced interconnectivity between the cerebral cortex and basal ganglia results in an impairment of motor functions [62], and this was evidenced via reduced performance in rodent behavioral tests. Treatment with CDCs or RSV improved motor performance and reduced PD symptoms associated with bradykinesia, rigidity, and postural control. Animals treated with neuroprotective agents displayed reduced catalepsy, reduction in abnormal behaviors and an overall improvement in movement, strength, speed, or balance [41,42,43,44,45,46,48,49,50,51,52,54,56,57]. 

#### 4.1.2. Anti-Oxidative Effects of Neuroprotective Agents

Neurons are vulnerable to oxidative damage but can mitigate redox stress through enzymatic and non-enzymatic defense mechanisms [63,64]. THC, BCP, and RSV displayed anti-oxidative effects via reduced ROS, DHBA, and NO production, with decreased MDA formation, lipid peroxidation, and PCC, and restoration of tricarboxylic acid cycle enzyme activities [43,44,46,47,48,50,51,54,56]. CDCs and RSV promoted upregulation of endogenous anti-oxidative enzymes and GSH levels to counter cellular redox stress, in part mediated via increased Nrf-2 activity [43,44,46,47,48,50,51,54]. However, Anandhan et al. [54] also reported reduced activity of SOD and CAT with RSV intervention. The polyphenolic structure of CBD, THC, THCV, and RSV provides a basis for free radical scavenging to mitigate oxidative stress, although interestingly, BCP was also still able to reduce the production of MDA [47], a marker of free radical damage to lipid bilayers. 

#### 4.1.3. Anti-Inflammatory Effects of Neuroprotective Agents

Neuroinflammation, mediated by activation of astrocytes and microglia, has a critical role in neurodegenerative diseases including PD [13,65,66], and this may in part relate to activation of human endogenous retroviruses [66]. Treatment with CDCs and RSV proved effective at reducing neuroinflammation in rodent PD models, as quantified by decreased levels of inflammatory protein markers and/or mRNA levels, as well as markers of activation of astrocytes and microglia [41,42,47,51,53,57]. The anti-inflammatory activity of CDCs and RSV may reflect activation of PPARs [33,34,67], and arise as a consequence of reduced oxidative stress [68,69].

#### 4.1.4. Anti-Apoptotic Effects of RSV

RSV displayed anti-apoptotic effects against nigral degeneration, with increased apoptotic markers Bcl-2 and pro-caspase 3 observed that paralleled decreased Bax and activated caspases [45,48,52]. The phosphoinositide 3-kinase (PI3K)/protein kinase B (Akt) signaling pathway was upregulated by RSV to reduce dopaminergic neuron injury [45]. Akt is involved in homeostatic regulation and is recruited to cell plasma membranes in response to cell stress, where it is phosphorylated by PDK1 at serine-437 and threonine-308 [70]. Akt activation can reduce Bax and activate caspase-3 levels to inhibit apoptosis. RSV treatment resulted in the upregulation of proteins involved in this pathway, and induced an increased p-Akt (ser437), PI3K-110α, and PDK-1 level, thus inhibiting neuronal apoptosis in PD rodents [45]. Consistent with a role for Akt, reduced p-Akt was detected in dopaminergic neurons from the SN in PD patients after analysis of *post-mortem* brain tissue [71].

In addition to anti-apoptotic effects, RSV reduced neuronal degradation by induction of autophagy of misfolded α-synuclein and p62 [52,72]. RSV treatment led to activated SIRT1, an NAD^+^-dependent deacetylase that deacetylates LC3-II intracellularly and results in increased cytoplasmic levels to break down α-synuclein aggregates [52,72,73]. Although not assessed by the captured references in this review, CDCs may also be cytoprotective via activation of autophagy [74].

Endoplasmic reticulum (ER) stress occurs in response to an imbalance in ER homeostasis as a result of a prolonged accumulation of misfolded or damaged proteins such as α-synuclein [75]. ER stress activates the unfolded protein response (UPR), and the apoptotic division of the UPR pathway contributes to the loss of striatal dopaminergic neurons in PD [76,77]. RSV treatment was able to reduce ER stress through downregulation of the glucose-regulated protein 78 (GRP78) and CCAAT/enhancer binding protein homologous protein (CHOP) [48,77]. GRP78 forms a complex with misfolded proteins, in turn initiating the UPR pathway. Overexpression of CHOP in ER stress stimulates the activation of the pro-apoptotic Bax protein facilitating activation of caspases. This may be one of the potential mechanisms influencing the beneficial decrease in apoptotic nigral cells following RSV treatment in rodent studies [44,45,48,52]. 

### 4.2. Pro-Dopaminergic Properties of CDC’s May Involve Cannabinoid Receptors

THC is a major cannabinoid constituent of the *Cannabis sativa* plant and interacts with the G-protein-coupled cannabinoid receptor 1 (CB1-R) and has a weak affinity for the cannabinoid receptor 2 (CB2-R) [78]. It might be expected that the attenuation of DA neuron loss in rodents would arise via stimulation of CB1 receptors in the CNS; however, a similar neuroprotective response was demonstrated using CBD [40], yet CBD has a low affinity for CB1 receptors. This suggests that the increase in TH-positive neurons and reduction in dopaminergic neuron loss could in part be mediated by a CB1-independent mechanism [40]. This was further supported by a demonstration that the minor phytocannabinoid THCV, which at low dose is a CB1R antagonist and a CB2R agonist, proved effective at countering a reduction in dopaminergic neuron levels with neuroprotective effects not attributed to CB1 binding, as a similar result was observed using a CBD derived drug [41]. Furthermore, the pro-dopaminergic action of THCV as a CB2 receptor agonist proved viable in an LPS-mediated inflammatory model of PD and demonstrated its anti-inflammatory potential for treatment in PD [41]. The stimulation of the CB1R by THC offers symptomatic relief against resting tremor in PD, but there are concerns regarding its ability to worsen hypokinesia and exhibit unwanted psychotropic effects [79]. In contrast, CBD is effective at attenuating dopaminergic neuron loss in a PD model [40] and may not induce the symptomatic complications associated with THC. Furthermore, the potentially beneficial immunomodulatory actions of cannabinoids such as CBD are associated with agonism to CB2 receptors [80]. Collectively, the neuroprotective effects of CBD may also be mediated via a number of non-endocannabinoid signaling pathways including G-protein-coupled receptors, a ligand-gated ion channel, as well as via PPAR-γ [81].

### 4.3. Clinical Trials Using Cannabinoids and RSV

Although there is a growing body of evidence from preclinical animal studies that support the neuroprotective effects of CDCs and RSV for PD, there remains a lack of corresponding human clinical trials that consider the effects of BCP, THC, THCV, or RSV. A search of human clinical trials (https://clinicaltrials.gov/ct2/home (accessed on: 21 November 2021) revealed only two studies that investigated the effects of CBD in PD, with study characteristics summarized in Table 8. An open-label dose-escalation study that investigated the efficacy of CBD in individuals with PD reported that CBD administration improved motor movement and sleep quality, evidenced via increased Movement Disorder Society-Unified Parkinson’s Disease Rating Scale (MDS-UPDRS) scores, with only mild adverse effects but with evidence of hepatotoxicity [82]. An exploratory double-blind trial investigating CBD reported no significant changes in movement scores, although there was an improvement in the Parkinson’s Disease Questionnaire (PDQ) scores and overall emotional well-being [83]. 

#### Bioavailability of CDCs and RSV

The efficacy of CDCs and RSV has been demonstrated in rodent studies and data from clinical trials has supported the potential safety of CBD and RSV in humans. Nonetheless, compounds such as RSV have limitations associated with relatively rapid metabolism and low bioavailability, although this may be combatted by acute dose escalation and/or more chronic dosing regimens, or utilization of derivatives of RSV [84,85,86,87,88]. 

Epidiolex, an oral solution of pure CBD has been approved therapeutically, but details regarding the pharmacokinetics and bioavailability of CBD are limited, with a report of 31% after smoking [89,90], however, when consumed orally, the bioavailability may only be as low as 6% [89,91]. Similarly, the oral bioavailability of THC is relatively low at 6% but increases to 27% when inhaled [92]. Sativex, a cannabis extract oromucosal spray that contains both ∆^9^-THC and CBD (and other cannabinoids) also has similar oral bioavailability to THC alone [93]. Due to the lipophilic nature of CBD, it is also often prescribed as an oromucousal spray or gel-encapsulation [89]. Moreover, the lipophilicity of CBD can be intentionally exploited when it is consumed, given that it can dissolve in high-fat nutrients to form micelle-structures favorable for gastrointestinal tract transport, thus increasing solubility and bioavailability [91]. However, whether these modifications result in increased bioavailability remains largely undetermined in humans, and also of concern is whether increased bioavailability impacts upon side effects, as short-term use of medicinal cannabinoids has a risk of non-serious adverse events and long-term use is as yet poorly characterized [94].

### 4.4. Study Limitations

A general limitation of the majority of PD models described is that the neurotoxins used to induce PD are fast acting and cause a relatively rapid depletion of dopaminergic neurons, typically within a period of several days, whereas in humans, disease progression is much slower, and may take two decades for dopaminergic neuron depletion and establishment of clinical sequelae. Thus, although the described models have proved useful in establishing pharmacological effects of CDCs and RSV to combat PD pathology, the acute action of the neurotoxins will not replicate a long-term disease course and the associated symptomology observed in humans. Therefore, one cannot predict the extent of the protective effects of CDCs or RSV in the early to moderate stages of PD. Indeed, an extension of the length of studies may prove useful to evaluate better the behavioral changes in motor and cognitive function in rodents, as well as increasing the validity of outcome measures. To that end, there may be a benefit to the use of genetic models of PD to establish subtle development of the disease over time.

In addition, individual variability with respect to patient age, co-morbidities, and genetic influences on disease progression cannot be anticipated using rodent models. Furthermore, although these neurotoxin models produce overlapping endpoints such as reduced dopamine and dopaminergic neuron levels that mirror the disease pathology, patients with PD also display non-motor symptoms such as sleep and psychiatric disorders and clinical overlap with patients with atypical Parkinsonian syndromes; complex sequelae that have yet to be suitably modelled in vivo. 

There is also a risk of experimental and publication bias in these pre-clinical animal trials. For example, a lack of details concerning blinding in allocation and outcome assessments. Furthermore, all studies cited in this review only performed experiments with male rodents, and there may well be sex-specific differences in PD pathology. Lastly, although the majority of studies employed oral and intragastric administration, particularly for RSV, some of the studies used subcutaneous, intraperitoneal, or intravenous administration routes, ones less likely to be adopted routinely by humans, and that will also influence compound bioavailability and pharmacokinetic data. 

## 5. Conclusions and Future Perspectives

To our knowledge, this is the first systematic review that has directly considered the effects of both selective CDCs and RSV in the neuroprotective treatment of PD. Collectively, in vivo rodent studies have demonstrated that these natural compounds are efficacious in their neuroprotection of PD and produced symptomatic benefits. However, there remains a need to expand these studies to more chronic models and ones able to better reproduce motor and non-motor symptoms of PD, and for additional studies that consider the benefits of formulations and derivatives of neuroprotective agents with improved bioavailability. Ultimately, further human clinical trials are required to consider the usefulness of neuroprotective agents for patients with early stage or early onset PD. 

## Figures and Tables

**Table 1:** Skeletal structures of the neuroprotective agents assessed in this review.

*trans*-Resveratrol (RSV)	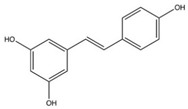
Cannabidiol (CBD)	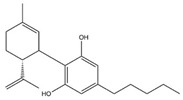
∆^9^-Tetrahydrocannabinol (THC)	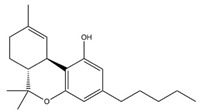
Tetrahydrocannabivarin (THCV)	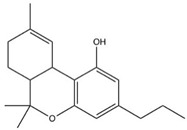
β-caryophyllene (BCP)	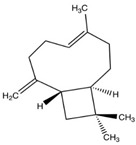

Structures taken from the pubchem.ncbi.nlm.nih.gov (accessed on 27 October 2021) database and drawn using ChemDraw.

**Table 2:** Study characteristics and outcome summary.

Author and Year	Species and Strain, Study Population	Neurotoxin Model, Dose, Route of Administration, Length of Dosing	Main Intervention Groups: Compound, Dose, Frequency	Outcomes in Main Interventional Groups
Ojha et al. (2016) [47]	Wistar rats. Four Groups (*n* = 8)	Rotenone induced (2.5 mg/kg bw per day, *i.p.*); 28 days	BCP (50 mg/kg bw per day dissolved in olive oil *i.p.*); 28 days	↓, DAN loss ↓, OS markers ↓, Inflammatory markers
Viveros-Paredes et al. (2017) [57]	C57BL/6J mice. Six groups (*n* = 6)	MPTP induced (30 mg/kg bw per day, *i.p.*); 5 days	BCP (10 mg/kg bw per day *i.p.*); 5 days. BCP (10 mg/kg bw per day *o.g.*); 5 days	↓, pole test time (s), ↑, stride length in gait test, ↓, time in beam test (s) ↑, TH–positive neurones ↓, inflammatory markers
Peres et al. (2016) [49]	Wistar rats. Four Groups (*n* = 10)	Reserpine induced (1 mg/kg bw per day, *s.c.*); 2 days	CBD (0.5 mg/kg and 5 mg/kg bw per day dissolved in saline and 1% Tween-80, *i.p.*); 3 days	↓, catalepsy
Lastres-Becker et al. (2005) [40]	SD rats. Four groups (*n* = 7+)	6-OHDA induced (8 μg *m.f.b.i.*); 14 days	CBD (3 mg/kg bw per day dissolved Tween-80:saline (1:16) *i.p.*); 14 days THC (3 mg/kg bw per day, dissolved in Tween-80:saline (1:16) *i.p.*); 14 days.	↑, T H mRNA
Abdel-Salam et al. (2012) [46]	SA mice. Seven groups (*n* = 6)	Haloperidol induced (1 mg/kg bw per day, *i.p.*); 18 days	Cannabis extract THC (10 mg/kg and 20 mg/kg bw per day, dissolved in 96% ethanol *s.c.*); 18 days	↓, catalepsy ↓, OS markers
Garcia et al. (2011) [41]	SD rats, CB2 −/− mice, wild-type littermates. Twelve groups (*n* = 5–6)	6-OHDA induced rats (200 μg per day, *i.c.v.*); 14 days. LPS induced mice (5 μg, *i.c.v.*); 14 days	THCV (2 mg/kg bw dissolved in Tween-80:saline (1:16) *i.p.*, single dose 14 days post-lesion.) THCV (2 mg/kg bw per day dissolved in Tween-80:saline (1:16) *i.p.*); 14 days	↑, activity in CAA test ↓, DAN loss, ↑, TH–positive neurons
Zhang et al. (2018) [51]	A53T α-synuclein mice, wild-type littermates. Five groups (*n* = 8)	A53T α-synuclein mouse model.	RSV (10 mg/kg and 50 mg/kg bw per day, *o.g.*; 35 days)	↓, activity in open field test, ↑, cognitive performance, ↓, hindlimb clasping, ↓, time pole test (s) ↓, α-synuclein ↓, OS markers ↓, inflammatory markers
Lu et al. (2008) [56]	Balb/C mice. Four groups (*n* = 15)	MPTP induced (30 mg/kg bw per day, *i.p.*); 7 days	RSV (20 mg/kg bw per day, dissolved in 20% ethanol *i.v.*); 7 days	↑, retention time on rotarod, ↓, grasp strength ↓, OS markers
Anandhan et al. (2010) [54]	Albino C57BL/6 mice. Four groups (*n* = 12)	MPTP induced (30 mg/kg bw per day *i.p.*); 4 days	RSV (50 mg/kg bw per day, *p.o.*); 7 days	↑, activity in open field test, ↑, retention time on the rotarod, ↓, time in beam test ↑, DA ↓, OS markers
Lofrumento et al. (2014) [53]	C57BL/6N mice. Four groups (*n* = 6)	MPTP induced (20 mg/kg bw, 4 doses, *i.p.*); 8 h	RSV (50 mg/kg bw per day, *o.g.*); 21 days	↓, apomorphine-induced circling behavior ↓, DAN loss ↓, inflammatory markers
Guo et al. (2016) [52]	C57BL/6 mice. Four groups (*n* = 12)	MPTP induced (30 mg/kg bw per day, *i.p.*); 5 days	RSV (100 mg/kg bw per day *i.g*.; 33 days)	↑, activity in open field test, ↑, catalepsy, ↑, rotarod performance ↑, TH–positive neurons ↓, pro-apoptotic markers
Xia et al. (2019) [55]	Mice. Three groups (*n* = 8)	MPTP induced (20 mg/kg bw, 4 doses, *i.p.*); 8 h	RSV (50 mg/kg bw per day, *i.g.*); 21 days	↑, TH–positive neurons, ↓, α-synuclein
Jin et al. (2008) [42]	SD rats. Six groups (*n* = 10)	6-OHDA induced. (10 μg *s.i.*)	RSV (10, 20 and 40 mg/kg in distilled water bw per day, *o.g.*); 70 days	↓, apomorphine-induced circling behavior ↓, DAN injury ↓, inflammatory markers
Khan et al. (2010) [43]	Wistar rats. Four groups (*n* = 8)	6-OHDA induced (10 μg *s.i.*)	RSV (20 mg/kg bw per day, dissolved in 20% ethanol, *i.p.*); 15 days	↓, apomorphine-induced circling behavior, ↑, performance in stepping test, ↑, retention time on the rotarod ↑, DA ↑, DOPAC ↓, OS markers
Wang et al. (2011) [44]	Wistar rats. Five groups (*n* = 10)	6-OHDA induced (15 μg *i.c.v.*)	RSV (1 mL, *g.g.*); 14 days RSV liposome (20 mg/kg bw per day, *i.g.*); 14 days	↓, apomorphine-induced circling behavior ↑, TH-positive neurons ↓, OS markers ↓, pro-apoptotic markers
Huang et al. (2019) [45]	SD rats. Five groups (*n* = 10)	6-OHDA induced (8 μg *i.p.*)	RSV (15 mg/kg and 30 mg/kg bw per day, *p.o.*); 36 days	↑, activity in open field test, ↑, catalepsy, ↑, rotarod performance ↑, TH–positive neurons ↓, pro-apoptotic markers
Gaballah et al. (2016) [48]	Wistar Albino rats. Four groups (*n* = 10–15)	Rotenone induced (1.5 mg/kg bw, 11 doses *s.c.*); 21 days	RSV (20 mg/kg bw per day, *o.g.*); 21 days	↓, catalepsy, ↑, retention time on rotarod ↑, DA ↓, OS markers ↓, inflammatory markers ↓, pro-apoptotic markers
Palle and Neerati (2018) [50]	Wistar Albino rats. Four groups (*n* = 12)	Rotenone induced (2 mg/kg bw per day *s.c.*); 35 days	RSV (40 mg/kg bw per day, *p.o.*) RSV nanoparticles (40 mg/kg bw per day, *p.o.*); 35 days	↑, rearing count ↑, rotarod performance ↓, OS markers

Abbreviations: BCP, β-caryophyllene; bw, bodyweight; CBD, cannabidiol; CAA, computer-aided actimeter; DA, dopamine; DAN, dopaminergic neurons; DOPAC, 3,4-Dihydroxyphenylacetic acid; *g.g.*, gastric gavage; 6-OHDA, 6-Hydroxydopamine; *i.c.v.*, intracerebroventricular cannulation; *i.g.*, intragastric gavage; *i.p.*, intraperitoneal; *i.v.*, intravenous; LPS, lipopolysaccharide; *m.f.b.i.*, median foramen bundle injection; MPTP, 1-methyl-4-phenyl-1,2,3,6-tetrahydropyridine; *o.g.*, oral gavage; OS, oxidative stress; *p.o.*, per os; RSV, resveratrol; SA, Swiss Albino; *s.c.*, subcutaneous; SD, Sprague Dawley; *s.i.*, striatal injection; TH, tyrosine hydroxylase; THC, ∆^9^-tetrahydrocannabinol; THCV, tetrahydrocannabivarin. ↑, denotes an increase; ↓, denotes a decrease.

**Table 3:** Summary of behavioral outcomes in PD model groups and interventions.

Author and Year	Intervention	Behavioral Outcomes in PD Model Group (vs. Controls)	Level of Significance	Behavioral Changes in Main Intervention Group (vs. PD Model Group)	Level of Significance
Viveros-Paredes et al. (2017) [57]	BCP	↑, pole test time (s) ↓, stride length in gait test ↑, time in beam test (s)	(*p* < 0.001) (*p* < 0.01) (*p* < 0.01)	↓, pole test time (s) ↑, stride length in gait test ↓, time in beam test (s)	(*p* < 0.01) (*p* < 0.05) (*p* < 0.01)
Peres et al. (2016) [49]	CBD	↓, activity in open field test ↑, catalepsy ↑, vacuous chewing	(*p* < 0.05)	↑, memory deficit but not locomotor activity in open field test ↓, catalepsy ↓, vacuous chewing	(*p* < 0.05)
Abdel-Salam et al. (2012) [46]	THC	↑, catalepsy	(*p* < 0.05)	↓, catalepsy	(*p* < 0.05)
Garcia et al. (2011) [41]	THCV	↓, activity in CAA test	(*p* < 0.05)	↑, activity in CAA test	(*p* < 0.05)
Jin et al. (2008) [42]	RSV	↑, apomorphine-induced circling behavior	-	↓, apomorphine-induced circling behavior	(*p* < 0.01)
Lu et al. (2008) [56]	RSV	↓, retention time on rotarod ↑, grasp strength	(*p* < 0.05)	↑, retention time on rotarod ↓, grasp strength	(*p* < 0.05)
Anandhan et al. (2010) [54]	RSV	↓, activity in open field test ↑, time in beam test ↓, retention time on rotarod	(*p* < 0.05)	↑, activity in open field test ↓, time in beam test ↑, retention time on the rotarod	(*p* < 0.05)
Khan et al. (2010) [43]	RSV	↑, apomorphine-induced circling behavior ↓, retention time on rotarod ↓, performance in stepping test	(*p* < 0.01) (*p* < 0.001) (*p* < 0.01)	↓, apomorphine-induced circling behavior ↑, retention time on the rotarod ↑, performance in stepping test	(*p* < 0.05) (*p* < 0.05) (*p* < 0.05)
Wang et al. (2011) [44]	RSV	↑, apomorphine-induced circling behavior	(*p* < 0.01)	↓, apomorphine-induced circling behavior	(*p* < 0.01)
Gaballah et al. (2016) [48]	RSV	↑, catalepsy ↓, retention time on rotarod	(*p* < 0.05)	↓, catalepsy ↑, retention time on rotarod	(*p* < 0.05)
Guo et al. (2016) [52]	RSV	↓, activity in open field test ↓, stride length in gait test ↑, time pole test (s)	(*p* < 0.001) (*p* < 0.001) (*p* < 0.01)	↑, activity in open field test ↑, stride length in gait test ↓, time pole test (s)	(*p* < 0.001) (*p* < 0.001) (*p* < 0.01)
Palle and Neerati (2018) [50]	RSV	↓, rearing count ↓, retention time on rotarod	(*p* < 0.05)	↑, rearing count ↑, retention time on rotarod	(*p* < 0.05)
Zhang et al. (2018) [51]	RSV	↑, activity in open field test ↑, time pole test (s) ↑, hindlimb clasping ↓, cognitive performance	(*p* < 0.05) (*p* < 0.001) (*p* < 0.001) (*p* < 0.05)	↓, activity in open field test ↓, time pole test (s) ↓, hindlimb clasping ↑, cognitive performance	(*p* < 0.05) (*p* < 0.01) (*p* < 0.05) (*p* < 0.05)
Huang et al. (2019) [45]	RSV	↓, retention time on rotarod ↓, activity in open field test ↑, catalepsy	(*p* < 0.05) (*p* < 0.01) (*p* < 0.05)	↑, rotarod performance ↑, activity in open field test ↓, catalepsy	(*p* < 0.05) (*p* < 0.01) (*p* < 0.05)

Abbreviations: BCP, β-caryophyllene; CAA, computer-aided actimeter; RSV, resveratrol; THC, ∆^9^-tetrahydrocannabinol. ↑, denotes an increase; ↓, denotes a decrease; “-” denotes not determined.

**Table 4:** Summary of biochemical and immunohistochemical analyses in PD model groups and interventions.

Author and Year	Intervention	Changes in Dopamine and α-Synuclein in PD Model Groups (vs. Control)	Level of Significance	Changes in Dopaminergic System and α-Synuclein in Main Interventional Groups (vs. PD Model)	Level of Significance
Ojha et al. (2016) [47]	BCP	↑, DAN loss	(*p* < 0.05)	↓, DAN loss	(*p* < 0.05)
Viveros-Paredes et al. (2017) [57]	BCP	↓, TH–positive neurons	(*p* < 0.001)	↑, TH–positive neurons	(*p* < 0.05)
Lastres-Becker et al. (2005) [40]	THC or CBD	↓, TH activity ↓, TH mRNA	(*p* < 0.05) (*p* < 0.01)	↑, TH activity ↑, TH mRNA	(*p* < 0.05) (*p* < 0.05)
Garcia et al. (2011) [41]	THCV	↑, DAN loss ↓, TH–positive neurons	(*p* < 0.05) (*p* < 0.005)	↓, DAN loss ↑, TH–positive neurons	(*p* > 0.05) (*p* < 0.05)
Jin et al. (2008) [42]	RSV	↑, DAN injury	-	↓, DAN injury	-
Anandhan et al. (2010) [54]	RSV	↓, DA ↓, DOPAC ↓, HVA	(*p* < 0.05)	↑, DA ↑, DOPAC ↑, HVA	(*p* < 0.05)
Wang et al. (2011) [44]	RSV	↓, TH-positive cells	(*p* < 0.05)	↑, TH-positive cells	(*p* < 0.01)
Lofrumento et al. (2014) [53]	RSV	↓, TH immunoreactivity	(*p* < 0.01)	↑, TH immunoreactivity	(*p* < 0.01)
Guo et al. (2016) [52]	RSV	↓, TH–positive neurons	(*p* < 0.001)	↑, TH–positive neurons	(*p* < 0.01)
Gaballah et al. (2016) [48]	RSV	↓, DA	(*p* < 0.05)	↑, DA	(*p* < 0.05)
Zhang et al. (2018) [51]	RSV	↑, α-synuclein ↑, A-11 positive oligomers ↑, W20 positive oligomers ↓, TH–positive neurons	(*p* < 0.001) (*p* < 0.001) (*p* < 0.001) (*p* < 0.01)	↓, α-synuclein ↓, A-11 positive oligomers ↓, W20 positive oligomers ↑, TH–positive neurons	(*p* < 0.001) (*p* < 0.05) (*p* < 0.05) -
Huang et al. (2019) [45]	RSV	↓, TH–positive neurons	(*p* < 0.05)	↑, TH–positive neurons	(*p* < 0.05)
Xia et al. (2019) [55]	RSV	↓, TH-positive neurons ↑, α-synuclein	(*p* < 0.05)	↑, TH–positive neurons ↓, α-synuclein	(*p* < 0.05)

Abbreviations: BCP, β-caryophyllene; DOPAC, 3,4-dihydroxyphenylacetic acid; DA, dopamine; DAN, dopaminergic neurons; HVA, homovanillic acid; RSV, resveratrol; TH, tyrosine-hydroxylase; THC, ∆^9^-tetrahydrocannabinol; THCV, tetrahydrocannabivarin. ↑, denotes an increase; ↓, denotes a decrease; -, denotes not determined.

**Table 5:** Summary of biochemical and immunohistochemical analysis for oxidative stress markers in PD model groups and interventions.

Author and Year	Intervention	Changes in Oxidative Stress in PD Model Group (vs. Control)	Level of Significance	Changes in Oxidative Stress Markers in Main Intervention Group (vs. PD Model Group)	Level of Significance
Ojha et al. (2016) [47]	BCP	↓, GSH ↑, MDA ↓, SOD and CAT	(*p* < 0.01)	↑, GSH ↓, MDA ↑, SOD and CAT	(*p* < 0.05)
Abdel-Salam et al. (2012) [46]	THC	↑, MDA ↑, NO ↓, GSH	(*p* < 0.05)	↓, MDA ↓, NO ↑, GSH	(*p* < 0.05)
Lu et al. (2008) [56]	RSV	↑, DHBA	(*p* < 0.05)	↓, DHBA	(*p* < 0.05)
Anandhan et al. (2010) [54]	RSV	↓, GSH ↑, SOD and CAT ↑, TBARS ↓, GPx	(*p* < 0.05)	↑, GSH ↓, SOD and CAT ↓, TBARS ↑, GPx	(*p* < 0.05)
Khan et al. (2010) [43]	RSV	↑, TBARS ↑, PCC ↓, GSH ↓, GPx ↓, GR ↓, SOD ↓, CAT	(*p* < 0.001) (*p* < 0.01) (*p* < 0.01) (*p* < 0.01) (*p* < 0.05) (*p* < 0.05) (*p* < 0.01)	↓, TBARS ↓, PCC ↑, GSH ↑, GPx ↑, GR ↑, SOD ↑, CAT	(*p* < 0.01) (*p* < 0.05) (*p* < 0.05) (*p* < 0.05) (*p* < 0.05) (*p* < 0.01) (*p* < 0.001)
Wang et al. (2011) [44]	RSV	↓, TAOC ↑, ROS	(*p* < 0.01)	↑, TAOC ↓, ROS	(*p* < 0.01)
Gaballah et al. (2016) [48]	RSV	↑, XO ↑, PCC ↓, GPx ↑, Nrf-2 DNA-binding activity	(*p* < 0.05)	↓, XO ↓, PCC ↑, GPx ↑, Nrf-2 DNA-binding activity	(*p* < 0.0001)
Zhang et al. (2018) [51]	RSV	↑, ROS ↑, MDA ↓, SOD and CAT	(*p* < 0.01)	↓, ROS ↓, MDA ↑, SOD and CAT	(*p* < 0.01)
Palle and Neerati (2018) [50]	RSV	↓, SDH ↓, CS ↓, aconitase ↓, MC-I activity ↓, GSH ↓, CAT ↑, MDA	(*p* < 0.05)	↑, SDH ↑, CS ↑, aconitase ↑, MC-I activity ↑, GSH ↑, CAT ↓, MDA	(*p* < 0.05)

Abbreviations: BCP, β-caryophyllene; CAT, catalase; CS, citrate synthase; DHBA, dihydroxybenzoic acid; GPx, glutathione peroxidase; GR, glutathione reductase; GSH, glutathione; MC-I, mitochondrial complex I; MDA, malondialdehyde; NO, nitric oxide; PCC, protein carbonyl content; ROS, reactive oxygen species; RSV, resveratrol; SDH, succinate dehydrogenase; SOD, superoxide dismutase; TAOC, total antioxidant capacity; TBARS, thiobarbituric acid reactive substances; THC, ∆^9^-tetrahydrocannabinol; XO, xanthine oxidase. ↑, denotes an increase; ↓, denotes a decrease.

**Table 6:** Summary of biochemical and immunohistochemical analysis for inflammatory mediators in PD model groups and interventions.

Author and Year	Intervention	Inflammatory Changes in PD Model Group (vs. Control)	Level of Significance	Inflammatory Changes in Main Intervention Group (vs. PD Model Group)	Level of Significance
Ojha et al. (2016) [47]	BCP	↑, GFAP ↑, Iba-1 ↑, IL-1β ↑, IL-6 ↑, TNF-α	(*p* < 0.05)	↓, GFAP ↓, Iba-1 ↓, IL-1β ↓, IL-6 ↓, TNF-α	(*p* < 0.05)
Viveros-Paredes et al. (2017) [57]	BCP	↑, GFAP-IR cells ↑, Iba-1-IR cells ↑, IL-1β ↑, IL-6 ↑, TNF-α	(*p* < 0.01) (*p* < 0.05) (*p* < 0.01) (*p* < 0.05) (*p* < 0.05)	↓, GFAP-IR cells ↓, IBA-1-IR cells ↓, IL-1β ↓, IL-6	(*p* < 0.01) (*p* < 0.01) (*p* < 0.01) (*p* < 0.05)
Garcia et al. (2011) [41]	THCV	↑, microglial activation	(*p* < 0.005)	↓, microglial activation	(*p* < 0.05)
Jin et al. (2008) [42]	RSV	↑, COX-2 ↑, TNF-α mRNA	(*p* < 0.01)	↓, COX-2 ↓, TNF-α mRNA	(*p* < 0.01)
Lofrumento et al. (2014) [53]	RSV	↑, GFAP mRNA expression ↑, CD11-immunoreactivity ↑, IL-1β mRNA ↑, TNF-α mRNA ↑, IL-6 mRNA ↑, IL-1β R1 ↑, TNF-α R1 ↑, IL-6Rα ↓, SOCS-1	(*p* < 0.01) (*p* < 0.01) (*p* < 0.01) (*p* < 0.01) (*p* < 0.05) (*p* < 0.05) (*p* < 0.05) (*p* < 0.01) (*p* < 0.01)	↓, GFAP mRNA expression ↓, CD11-immunoreactivity ↓, IL-1β mRNA ↓, TNF-α mRNA ↓, IL-6 mRNA ↓, IL-1β RI ↓, TNF-α RI ↓, IL-6Rα ↑, SOCS-1	(*p* < 0.01) (*p* < 0.05) (*p* < 0.01) (*p* < 0.05) (*p* < 0.01) (*p* < 0.05) (*p* < 0.05) (*p* < 0.05) (*p* < 0.01)
Gaballah et al. (2016) [48]	RSV	↑, striatal IL-1β levels	(*p* < 0.05)	↓, striatal IL-1β levels	(*p* < 0.05)
Zhang et al. (2018) [51]	RSV	↑, GFAP ↑, Iba-1 ↑, IL-1β ↑, IL-6 ↑, TNF-α	(*p* < 0.001) (*p* < 0.001) (*p* < 0.01) (*p* < 0.001) (*p* < 0.05)	↓, GFAP ↓, Iba-1 ↓, IL-1β ↓, IL-6 ↓, TNF-α	(*p* < 0.05) (*p* < 0.01) (*p* < 0.05) (*p* < 0.05) (*p* < 0.01)

Abbreviations: BCP, β-caryophyllene; COX-2, cyclooxygenase-2; GFAP, glial fibrillary acidic protein; GFAP-IR, glial fibrillary acidic protein immunoreactive cells; Iba-1, ionized calcium-binding adaptor molecule-1; Iba-1-IR, ionized calcium-binding adaptor molecule immunoreactive cells; IL-1β, interleukin-1 beta; IL-1β R1, interleukin-1 beta receptor 1; IL-6, interleukin-6; IL-6Rα, interleukin-6 receptor alpha; RSV, resveratrol; TNF-α, tumor necrosis factor-alpha; TNF-α R1, tumor necrosis factor-alpha receptor 1; SOCS-1, suppressor of cytokine signaling 1. ↑, denotes an increase; ↓, denotes a decrease.

**Table 7:** Summary of biochemical and immunohistochemical analysis for markers of apoptosis PD model groups and interventions.

Author and Year	Intervention	Apoptotic Changes in PD Model Group (vs. Control)	Level of Significance	Apoptotic Changes in Main Intervention Group (vs. PD Model Group)	Level of Significance
Wang et al. (2011) [44]	RSV	↓, apoptotic nigral cells	(*p* < 0.01)	↓, apoptotic nigral cells	(*p* < 0.01)
Gaballah et al. (2016) [48]	RSV	↑, CHOP and GRP78 ↑, striatal caspase-3 activity	(*p* < 0.05)	↓, CHOP and GRP78 ↓, striatal caspase-3 activity	(*p* < 0.05)
Guo et al. (2016) [52]	RSV	↑, cleaved caspase 3 ↓, deacetylated LC3-II ↑, p62	(*p* < 0.001) (*p* < 0.001) (*p* < 0.01)	↓, cleaved caspase 3 ↑, deacetylated LC3-II ↓, p62	(*p* < 0.01) (*p* < 0.001) (*p* < 0.001)
Huang et al. (2019) [45]	RSV	↑, Bax ↑, activated caspase 3 ↓, Bcl-2 ↓, Pro-caspase-3 expression	(*p* < 0.01) (*p* < 0.01) (*p* < 0.05) (*p* < 0.01)	↓, Bax ↓, activated caspase 3 ↑, Bcl-2 ↑, Pro-caspase-3 expression	(*p* < 0.01) (*p* < 0.01) (*p* < 0.05) (*p* < 0.01)

Abbreviations: Bax, Bcl-2-associated X protein; Bcl-2, B-cell lymphoma 2; CHOP, C/EBP homologous protein; GRP78, glucose regulated protein 78; LC3-II, microtubule-associated protein 1A/1B-light chain 3; RSV, resveratrol. ↑, denotes an increase; ↓, denotes a decrease.

**Table 8:** Summary of the characteristics of clinical human trials investigating the use of CBD in patients with PD.

Author & Year	Study Type	Population	Intervention	Study Outcome
Leehey et al. (2020) [82] NCT02818777	Safety and tolerability of CBD in PD, open label dose-escalation study	13 participants, mean age 68 (SD = 6) with PD	CBD: (Epidiolex; 100 mg/mL); 5 to 20–25 mg/kg/day; 10–15 days	Improved MDS-UPDRS scores. Improved sleep and emotional dyscontrol scores. Mild adverse effects including diarrhea, somnolence, fatigue, weight gain, dizziness, abdominal pain, weight loss, headache, nausea, anorexia, increased appetite. Increase liver enzyme profile, cholestatic in nature.
Chagas et al. (2014) [83] NCT unavailable	CBD as a treatment for patients with PD, exploratory double-blind trial	119 individuals; PD	CBD; 75 mg/day or CBD 300 mg/day; 36 days	No significant changes in UPDRS scores, plasma BDNF levels or H^1^-MRS measures. Improved PDQ scores and overall emotional well-being.

Abbreviations: BDNF, brain-derived neurotrophic factor; CBD, cannabidiol; H^1^-MRS, proton magnetic resonance spectroscopy; MDS-UPDRS, Movement Disorder Society-Unified Parkinson’s Disease Rating Scale; PD, Parkinson’s disease; PDQ, Parkinson’s Disease Questionnaire; SD, standard deviation.

## Supplementary Materials

The following are available online at https://www.mdpi.com/article/10.3390/brainsci11121573/s1, Figure S1: Annual frequency of publications regarding PD and certain dietary polyphenols, Data S2: Electronic data search parameters and methodological quality assessment, Table S1: Risk of bias of included studies.
