# Supplementary material for: The Neuroprotective Effects of Cannabis-Derived Phytocannabinoids and Resveratrol in Parkinson’s Disease: A Systematic Literature Review of Pre-Clinical Studies
Source: Brain Sci. 2021 Nov 28;11(12):1573. doi: 10.3390/brainsci11121573 (PMC8699487; doi:10.3390/brainsci11121573)
Supplement: Supplementary file 1 [file brainsci-11-01573-s001.zip › brainsci-1459485-supplementary.pdf]

brain sciences

MDPI

Systematic Review

The Neuroprotective Effects of Cannabis-Derived Phytocannabinoids and Resveratrol in Parkinson's Disease: A Systematic Literature Review of Pre-Clinical Studies

Samay Prakash and Wayne G. Carter *

Royal Derby Hospital Centre, School of Medicine, University of Nottingham, Derby DE22 3DT, UK; mzysp8@nottingham.ac.uk
* Correspondence: wayne.carter@nottingham.ac.uk; Tel.: +44-(0)-1332-724738; Fax: +44-(0)-1332-724626

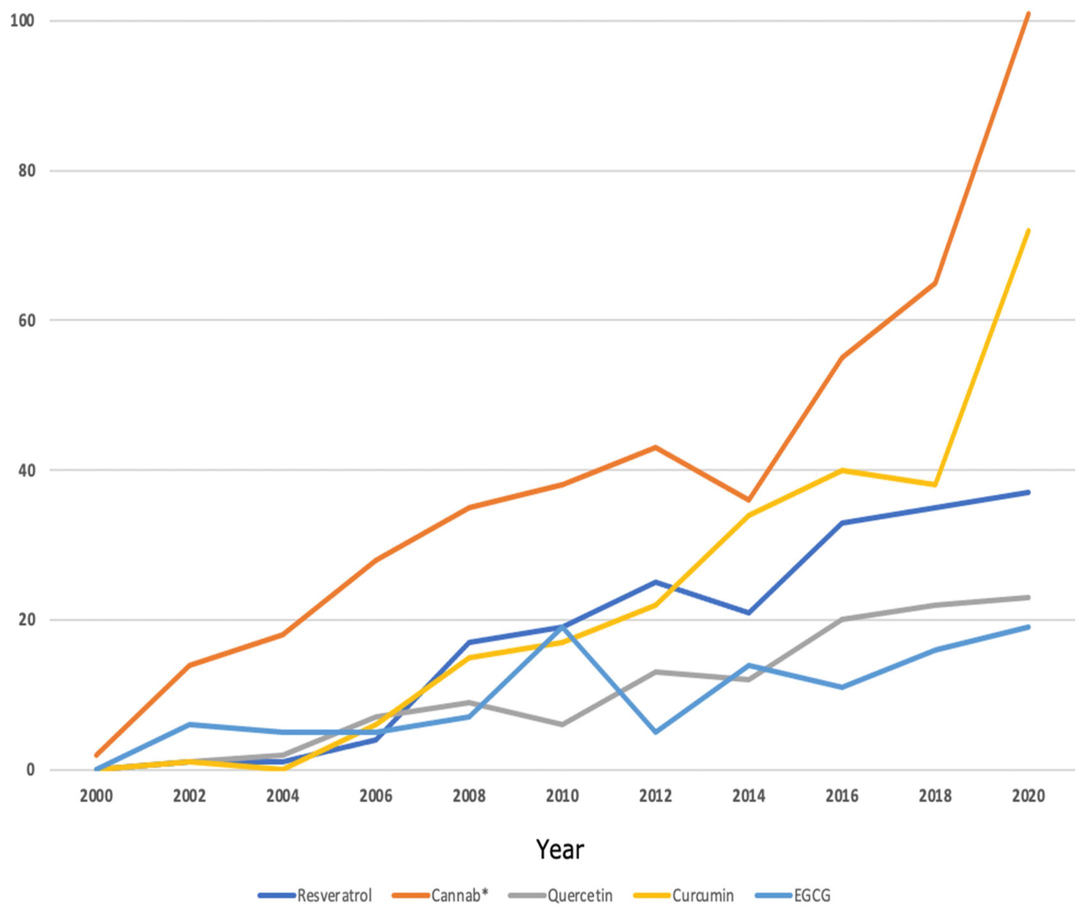

Supplementary Figure S1. Annual frequency of publications regarding PD and certain dietary polyphenols. Data generated according to a PubMed literature search (performed by the manuscript author (Samay Prakash) November 2020, using the search terms (PD and resveratrol, PD and cannab*, PD and quercetin, PD and curcumin, and PD and EGCG (epigallocatechin gallate).

Supplementary Data S2. Electronic data search parameters.

An electronic literature search was performed using: (1) Medline (OvidSP), (2) Embase (OvidSP), (3) APA PsychINFO (OvidSP), (4) PubMed and (5) Web of Science Core Collection. The following search terms were used in selected literature databases to uncover relevant publications regarding the neuroprotective effects of CDCs and RSV, within *in vivo* pre-clinical studies observing PD. Boolean operators were used to broaden the search results to incorporate all potentially relevant

research. Field tags such as: topic (TS), title (TI), multipurpose (m.p.) were used for more specific identification of key terms in titles and abstracts etc.

Ovid - Medline, Embase and APA PsychINFO:

1. Parkinson's Disease **OR** Parkinson*.mp.
2. Cannab* **OR** Tetrahydrocannabinol **OR** Cannabidiol **OR** β-Caryophyllene **OR** Tetrahydrocannabivarin **OR** Resveratrol.mp.
3. *in vivo* **OR** pre-clinical.mp.
4. Animal **OR** Primate **OR** Monkey **OR** Rodent **OR** Mice **OR** Mouse **OR** Rat.mp.
5. #1 **AND** #2 **AND** #3 **AND** #4

PubMed:

(((Parkinson's Disease OR Parkinson*)) **AND** ((Cannab* **OR** Tetrahydrocannabinol **OR** Cannabidiol **OR** β-Caryophyllene **OR** Tetrahydrocannabivarin **OR** Resveratrol)))

Web of Science:

1. **Topic (TS)** = (Parkinson's Disease **OR** Parkinson*)
2. **Title (TI)** = (Parkinson's Disease **OR** Parkinson*)
3. #1 OR #2
4. **TS** = (Cannab* **OR** Tetrahydrocannabinol **OR** Cannabidiol **OR** β-Caryophyllene **OR** Tetrahydrocannabivarin **OR** Resveratrol)
5. **TI** = (Cannab* **OR** Tetrahydrocannabinol **OR** Cannabidiol **OR** β-Caryophyllene **OR** Tetrahydrocannabivarin **OR** Resveratrol)
6. #4 OR #5
7. **TS** = (Animal **OR** Primate **OR** Monkey **OR** Rodent **OR** Mice **OR** Mouse **OR** Rat)
8. **TI** = (Animal **OR** Primate **OR** Monkey **OR** Rodent **OR** Mice **OR** Mouse **OR** Rat)
9. #7 OR #8
10. #3 **AND** #6 **AND** #9

The search term 'cannab*' was used to incorporate cannabis, cannabigerol, cannabidiols and all other cannabis-derived phytocannabinoids (CDCs).

Methodological Quality Assessment

Currently, there is no official gold standard tool for assessing bias in pre-clinical animal studies. Instead, the SYRCLE's risk of bias tool has been developed considering aspects of the 'Cochrane Collaboration risk of bias tool' used in randomised controlled trials [31]. The tool has been adapted in accordance with methodology used in animal studies. The following letters (A-J) indicate which specific aspect of the methodology is being assessed.

A: Was the allocation sequence adequately generated and applied? (Selection bias)
B: Were the groups similar at baseline or were they adjusted for confounders in the analysis? (Selection bias)
C: Was the allocation adequately concealed? (Selection bias)
D: Were the animals randomly housed during the experiment? (Performance bias)
E: Were the caregivers and/or investigators blinded from knowledge of which intervention each animal received during the experiment? (Performance bias)
F: Were animals selected at random for outcome assessment? (Detection bias)
G: Was the outcome assessor blinded? (Detection bias)
H: Were incomplete outcome data adequately addressed? (Attrition bias)
I: Are reports of the study free of selective outcome reporting? (Reporting bias)
J: Was the study apparently free of other problems that could result in high risk of bias? (Other)

Supplementary Table S1. Risk of bias of included studies.

Study	A	B	C	D	E	F	G	H	I	J
Ojha et al. (2016) [39]	N	Y	N	Y	UC	UC	Y	UC	Y	Y
Viveros-Paredes et al. (2017) [49]	Y	Y	N	Y	UC	UC	N	UC	Y	Y
Peres et al. (2016) [41]	N	Y	N	Y	UC	UC	Y	UC	Y	Y
Lattress Becker et al. (2005) [32]	N	Y	N	Y	UC	UC	N	UC	Y	Y
Abdel-Salam et al. (2012) [38]	N	Y	N	Y	UC	UC	N	UC	Y	Y
Garcia et al. (2011) [33]	N	Y	N	Y	UC	UC	Y	UC	Y	Y
Zhang et al. (2018) [43]	N	Y	N	Y	UC	UC	Y	UC	Y	Y
Lu et al. (2008) [48]	Y	Y	N	Y	UC	UC	N	UC	Y	Y
Anandhan et al. (2010) [46]	N	Y	N	Y	UC	UC	Y	UC	Y	Y
Lofrumento et al. (2014) [45]	N	Y	N	Y	UC	UC	N	UC	Y	Y
Guo et al. (2016) [44]	Y	Y	N	Y	Y	UC	Y	UC	Y	Y
Xia et al. (2019) [47]	N	Y	N	Y	UC	UC	N	UC	Y	Y
Jin et al. (2008) [34]	Y	Y	N	Y	UC	UC	N	UC	Y	Y
Khan et al. (2010) [35]	N	Y	N	Y	UC	UC	N	UC	Y	Y
Wang et al. (2011) [36]	Y	Y	N	Y	UC	UC	N	UC	Y	Y
Huang et al. (2019) [37]	Y	Y	N	Y	UC	UC	UC	UC	Y	Y
Gaballah et al. (2016) [40]	Y	Y	N	Y	UC	UC	N	UC	Y	Y
Palle et al. (2018) [42]	Y	Y	N	Y	UC	Y	N	UC	Y	Y

*Abbreviations: Y, Yes, low risk of bias; UC, Unclear, insufficient information to assess certain bias; N, No, high risk of bias.
